# Omics-Based Identification of Biomarkers for Nasopharyngeal Carcinoma

**DOI:** 10.1155/2015/762128

**Published:** 2015-04-27

**Authors:** Tavan Janvilisri

**Affiliations:** Department of Biochemistry, Faculty of Science, Mahidol University, Bangkok 10400, Thailand

## Abstract

Nasopharyngeal carcinoma (NPC) is a head and neck cancer that is highly found in distinct geographic areas, such as Southeast Asia. The management of NPC remains burdensome as the prognosis is poor due to the late presentation of the disease and the complex nature of NPC pathogenesis. Therefore, it is necessary to find effective molecular markers for early detection and therapeutic measure of NPC. In this paper, the discovery of molecular biomarker for NPC through the emerging omics technologies including genomics, miRNA-omics, transcriptomics, proteomics, and metabolomics will be extensively reviewed. These markers have been shown to play roles in various cellular pathways in NPC progression. The knowledge on their function will help us understand in more detail the complexity in tumor biology, leading to the better strategies for early detection, outcome prediction, detection of disease recurrence, and therapeutic approach.

## 1. Introduction

Nasopharyngeal carcinoma (NPC) is a cancer of the head and neck region that arises from the squamous epithelial cells covering the surface of nasopharynx, the uppermost part of the pharynx extending from the base of the skull to the upper surface of the soft plate [1]. The incidence of NPC varies greatly on the basis of ethnic and geographical backgrounds. While NPC is a rare malignancy in most parts of the world, it is one of the most common cancers in the East and Southeast Asia including China, Hong Kong, Taiwan, Singapore, Malaysia, and Thailand [2]. The annual incidence of NPC in the United States is about 5 per 100,000. The annual incidence of the NPC in the southern part of China including Taiwan is more than 10 per 100,000 and is up to 30 per 100,000 in Hong Kong. The annual incidence of the NPC in Southeast Asia such as Malaysia and Thailand is ~20 per 100,000 and ~7 per 100,000, respectively [2]. The etiological factors for NPC include the Epstein-Barr virus (EBV) infection, ethnics, genetic susceptibility, environmental factors, and consumption of food with volatile nitrosamines [3, 4].

NPC can be diagnosed and staged by a biopsy of the tissue mass, together with positron emission tomography (PET) and computed tomography (CT). However, most of NPC patients tend to present at a more advanced stage of the disease because the primary anatomical site of tumor growth is located in the silent painless area. Moreover, NPC in advanced stages exhibits higher metastatic potential than other head and neck squamous cell carcinomas [5]. On the basis of local anatomic constraints of NPC and its tendency to present with cervical lymph node metastasis, surgery has no role for definitive therapy. At present, radiotherapy represents the standard treatment for NPC. The disease tends to be more sensitive to radiation than other cancers, but the success depends mostly on the tumors stages, which tend to be in the advanced stages at the point of diagnosis. The 5-year survival rate of stages I and II NPC ranges from 72 to 90%. However, the 5-year survival rates of stages III and IV NPC are ~55% and 30%, respectively, due to a relatively high incidence of locoregional recurrence or metastasis [6]. In case of advanced tumors, both regional-control and distant metastatic tumors, the patients are usually treated with systemic therapy. Concurrent chemotherapy is generally accepted to have a role in management of locally advanced disease. The combination chemotherapy has been used with concurrent cisplatin and radiation followed by adjuvant cisplatin and 5-fluorouracil [7, 8].

**Figure 1:**
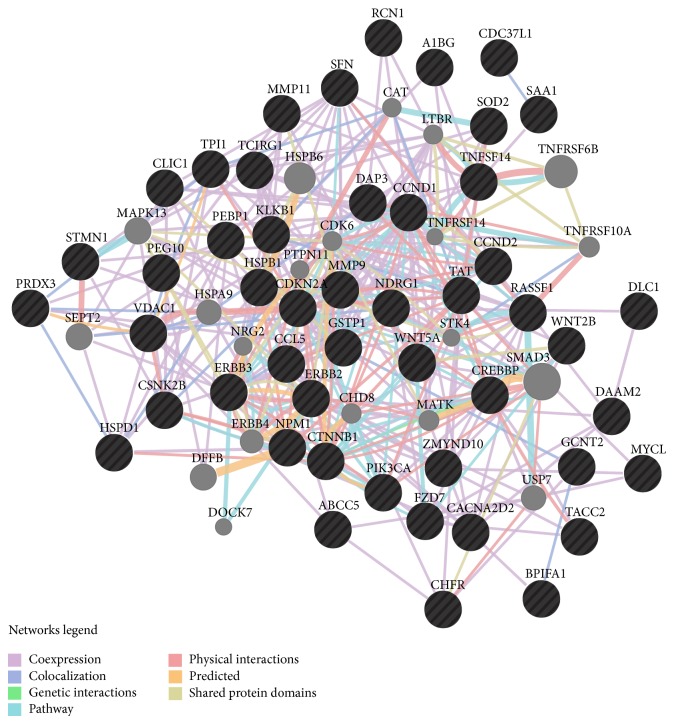
A network map of molecular biomarkers for nasopharyngeal carcinoma identified through omics technologies. Detailed information is described in the text.

NPC patients mostly appear in advanced stages of the disease and have a poor prognosis because of late presentation of lesions, limited knowledge of molecular pathogenesis, lack of reliable and robust biomarkers for early detection, and poor response to available therapies [9]. One of the reasons for the lack of effective molecular markers is that NPC is a highly complex multifactorial disease caused by an interaction of host genetics with the macro- and microenvironment that is influenced by EBV chronic infection and other environmental factors, in a multistep process of tumorigenesis [10]. In-depth understanding of the molecular alterations in and across the cellular pathways involved in NPC carcinogenesis can certainly facilitate the integration of diagnosis, anticancer drug discovery, and therapy for NPC. In the postgenomics era, an exponential growth of our knowledge on the disease etiology, carcinogenesis, and progression has been gained through an adoption of high-throughput technologies including genomics, transcriptomics, proteomics, metabolomics, and bioinformatics together with integration and application of systems biology. An increasing mass of these omics data has leads us to identify potential molecular targets for diagnosis, prognosis, and therapeutic treatment. The scope of this review is to shed light on the current findings of NPC biomarker discovery through the omics approaches. An overview of NPC biomarkers identified through omics approaches described herein is illustrated in Figure 1 using GeneMania (http://www.genemania.org/).

## 2. Genomics

Biomarkers at the genomic level can be retrieved by comparative genomic hybridization (CGH), exome sequencing, and whole genome sequencing. These biomarkers can identify genomic alterations including single-nucleotide polymorphisms (SNPs), copy number variations (CNVs), and other structural variations in the genome and may have functional significance in the pathophysiology of a defined phenotype. As genomic instability including amplification of oncogenes and/or deletion of tumor suppressor genes, together with dysfunction of the gene by point mutations, can be an early event marker in carcinogenesis of NPC, and there are several CGH studies to analyze the gain and the loss of genetic materials in the genome. Chen and colleagues [11] performed CGH on a total of 51 NPC cases including 25 primary and 26 recurrent tumors. They reported the chromosomal hotspots for copy number gains including the chromosome arms 12p, 1q, 11q, 12q, and 17q and losses including 3p, 9p, 11q, 13q, and 14q. They also showed that there was no additional chromosomal alteration in the recurrent tumors compared to the primary cancers. A few other studies based on CGH approach have also been reported [12–15]. The patterns of genomic imbalances in NPC from these CGH data appeared to be largely consistent with those identified in banding analysis and loss of heterozygosity studies. However, the discrepancy between these studies exists, which may be due to the different cohorts of samples with different clinicopathological backgrounds, reflecting variations in distinct types of carcinogens to the oncogenic process. Furthermore, the existing CGH data of 103 NPC cases were integrated and input into evolutionary tree models, which revealed the chromosomal loss of 3p and the gain of chromosome 12 as an important hallmark for an early event for NPC carcinogenesis [16].

In the past decade, microarray technology has served as an essential tool for examination of genetic profiles of biological samples and enables us to analyze more than ten thousand genes at a time, which can reveal genetic abnormalities in cancers at a genome-wide level. The principle of microarray is based on the complementary hybridization between nucleotide chains such as DNA-to-RNA strands and DNA-to-DNA strands [17]. A microarray is basically a microscopic slide with up to hundreds of thousands of DNA fragments, which are dotted on its surface with ~50–150 *μ*m diameter. The fragments are robotically printed or synthesized* in situ*. Each DNA fragment has a corresponding complementary DNA that binds to it. The genomic DNA can be isolated from tumor and/or normal samples, which can then be labeled with fluorophores such as cyanine-3 (Cy3; green) and cyanine-5 (Cy5; red) prior to hybridization. These labeled DNAs are added to the slide and thousands of hybridization reactions occur between input DNA samples and DNA probes on the microarray slides. After microarray laser scanning, the fluorescence values at each spot reveal the relative levels of copy number of the corresponding region [18]. Array-based CGH has therefore been used extensively to detect and quantify genomic aberrations in NPC and map onto chromosomal positions to identify relevant oncogenes or tumor suppressor genes. Hui and coworkers [19] utilized array-CGH to simultaneously investigate amplification of 58 oncogenes throughout the genome of 15 NPC samples including five cell lines, two xenografts, and eight primary tumours. The frequency of oncogenes including* MYCL1*,* TERC*,* ESR*, and* PIK3CA* were found to be amplified in the NPC samples. Other array-CGH experiments on different NPC samples have also been reported and revealed similar geographic variations in the frequencies of chromosome aberrations [15, 20, 21].

Recently, a genome-wide analysis of chromosome copy number was performed in the C666-1 cell line and from 15 NPC biopsies using high-density microarrays [22]. The data are in broad agreement with the data from conventional CGH, in which the copy loss at 3p, 9p, and 11q was observed. It has been revealed that several tumor suppressor genes such as* CDKN2A*,* ZMYND10*,* RASSF1*,* NDRG1*,* TACC2*, and* CACNA2D2* are significantly enriched within genomic regions that are frequently deleted; however, no significant correlation is established between the presence of potential tumor promoting genes and the genomic regions exhibiting gain of copy number [22]. As aberrant DNA methylation has been recognized to be associated with the transcriptional inactivation of genes related to cancer development, the application of microarrays has also been extended to study genome-wide DNA methylation patterns in NPC. Zhang and collaborators [23] investigated the methylation alterations in the genome of taxol-resistant NPC cell lines. The differential methylation profiles between the taxol-sensitive and taxol-resistant cells have been demonstrated, where the global hypermethylation was found in the latter case. The hypermethylated genes, namely,* DLC1*,* PEG10*, and the hypomethylated genes, namely,* ABCC5*,* CHFR*,* ERBB2*, and* GSTP1*, were identified and confirmed as downregulated and upregulated, respectively, in the resistant cells. Yang and colleagues [24] also applied the microchip containing ~27 k CpG loci covering more than 14,000 genes at single-nucleotide resolution to evaluate the effect of trichostatin A, one of the most potent HDAC inhibitors, on genome-wide DNA methylation pattern of a NPC cell line CNE2. Their data showed that the DNA methylation in trichostatin A-treated cells appeared to be higher in total compared to the controls. The hypermethylation of genes, namely* DAP3*,* HSPB1,* and* CLDN,* was identified in the treated group and the results were validated through quantitative reverse transcription polymerase chain reaction to confirm them as downregulated genes upon the treatment.

The discovery of variations in the DNA sequence of tumor cells associated with clinical significance has been hurled ahead by next-generation sequencing technologies. A combination of whole-exome and targeted deep sequencing, as well as SNP array analysis has been applied in order to characterize the mutational landscape of 128 NPC cases [25]. The results revealed multiple recurrent copy number variations with the most frequent deletion region covering the gene* CDKN2A* on 9p21. The loss of this chromosomal region has also been identified in the conventional CGH [11–15], providing further support for this NPC hotspot. Differential copy numbers in the genes, namely,* CCND1*,* AKT2*,* MYC*, and* TP53,* have also been observed. Interrogating pathway analyses also highlighted the dysregulation of cellular pathways involving in chromatin modification and ERBB-PI3K signaling pathway. Furthermore, the data indicated that the alterations in ERBB-PI3K pathway were linked to the more advanced stages and the survival of NPC patients with ERBB-PI3K mutations was shorter than the patients without such mutations [25]. Our recent work also supported this notion, where we demonstrated the abnormal expression of ERBB proteins and showed that the expression of ERBB3 was associated with patient survival and could serve as a novel and valuable predictor for prognostic evaluation of patients with NPC [26].

## 3. Transcriptomics

Expression biomarkers are traditionally derived through the measurement of a single gene or a cluster of biochemical and histopathological molecules in a given pathway. Transcriptomics or gene expression profiling offers evaluation of the levels of gene expression of all transcripts in a given sample at the same time. The conceptual idea of transcriptomics is that the genes involved in a particular pathophysiology often function in a concerted fashion and therefore the genes with similar expression patterns may be functionally associated and/or under similar molecular regulation [27]. Initially, suppression subtractive hybridization has been applied on a cohort of libraries of PCR-amplified cDNA fragments that differ between control (normal) and experimental (cancer) transcriptome [28]. Zhang and coworkers constructed the human embryo nasopharynx cDNA library in order to isolate and screen tissue-specific genes of human nasopharynx and new tumor suppressor genes of NPC [29, 30]. Microarray-assisted analysis of subtracted cDNA libraries constructed by suppression subtractive hybridization has been performed to search for differentially expressed genes and screen candidate molecular markers in NPC [31]. The differential transcriptomes of 9 NPC cases, 3 NPC cell lines, and 10 chronic inflammation of nasopharyngeal mucosa tissue samples and the result validation using real-time quantitative reverse transcription polymerase chain reaction and* in situ* hybridization techniques revealed that the palate, lung, and nasal epithelium carcinoma (*PLUNC*) and* Homo sapiens* cell division cycle 37 Homo sapien cell division cycle 37 homolog (*Saccharomyces cerevisiae*)-like 1 (*CDC37L1*) might serve as the potential molecular biomarkers for NPC [31].

High-throughput technologies based on the well-established DNA microarray represent the most cost-effective and convenient means to assess the gene expression profiles. However, a number of biological replicates or samples of the same condition as well as additional validation through qRT-PCR are necessary to eventually identify the biomarkers for class prediction on an independent validation set as the true changes in gene expression are often underestimated [32]. As the chronic EBV infection poses as one of the causative risk factors for NPC, the application of the microarray platform to distinguish transcriptome of the EBV− and EBV+ NPC cells has enabled us to gain more understanding of EBV-specific signals for NPC tumorigenesis [33]. A set of EBV-regulated genes has been identified, involved in cellular processes such as cell proliferation, cell cycle control, and cell mobility [33]. Because NPC tissue is heterogeneous comprising cancer cells, infiltrating inflammatory cells, and nonneoplastic nasopharyngeal epithelium and stroma, tissue microdissection of NPC and normal epithelial nasopharynx has been applied to select specific types of cells on the slides prior to being subjected to the gene expression profiling analysis. Collectively, these data point to the differential NPC genes involving in the cell cycle, apoptosis, tumor suppressors, cell adhesion, and motility [34–37]. The Wnt pathways, such as wingless-type MMTV integration site family, member 5A (WNT5A), FZD7, casein kinase II*β* (CSNK2B), *β*-catenin (CTNNB1), CREB-binding protein (CREBBP), and dishevelled-associated activator of morphogenesis 2 (DAAM2), transforming growth factor *β* (TGF*β*), and mitogen-activated protein kinase signaling pathway, have been found to be induced in NPC [35, 38]. Furthermore, among these genes, cyclin D1 has been shown to be the prognostic biomarker for NPC patients [37].

Microarray technology has also been used to explore the biological functions of novel genes in NPC at different metastatic features, clinical stages, and aggressive states. According to the metastatic states, the comparison of global gene expression patterns in NPC cells lines 5–8F (high tumorigenic and metastatic) and 6–10B (low tumorigenic and metastatic) revealed a cohort of genes involving in cell cycle, apoptosis, metastasis, chemokine, and immunomodulation, which potentially mediate their differential metastatic characteristics. Among them, PTHLH has been suggested to regulate the WNT pathway through the* DKK1* gene to affect metastasis and the apoptosis processes of NPC [39]. However, the validation in an independent set of samples is required to confirm this finding. Su et al. [40] identified a number of transcription factors including ATF1 and ATF2 to be associated with clinical stages. The potential downstream molecules for these transcription factors include the epithelial growth factor receptor (EGFR/ERBB1) and matrix metalloproteinase 2 (MMP-2). As the main pathological type of NPC appears to be nonkeratinizing carcinoma, gene expression profile changes have been evaluated among differentiated-type nonkeratinizing NPC cases, which revealed possible molecular subtypes [41]. It has been shown that the expression of cyclin D2 (CCND2) could serve as a molecular marker for the more aggressive tumor subtype and a strong predictor for survival time in this group of NPC patients.

The more recent RNA sequencing (RNASeq) approach utilizes deep-sequencing technologies to identify differential expression of an entire genome at any specific sample in any given time point, albeit rather expensive at present [42]. Szeto and colleagues characterized the transcriptomes of undifferentiated EBV-positive NPC xenograft X666 and its derived cell line C666, well-differentiated NPC cell line HK1, and the immortalized nasopharyngeal epithelial cell line NP460 using Solexa sequencing [43]. A total of 2,812 differentially expressed genes were identified among these samples and together with gene enrichment analysis, the extracellular matrix organization, beta-1 integrin cell surface interactions, and the PI3K/AKT, ERBB, and Wnt pathways were dysregulated in NPC [43]. In agreement with these findings, comparison of the gene expression of tumor cells and normal controls in recent studies also revealed that the Wnt, PI3K/AKT, and ERBB signalling pathways were dysregulated [22, 25].

A large number of NPC gene expression profiles have emerged in public databases. It is challenging to integrate these data from several datasets to yield maximal information. Researchers have employed meta-analysis of transcriptomic data by integrating them from multiple studies to successfully identify new prognostic and diagnostic markers for cancer and other diseases [44]. It involves a systematic search for proper datasets and data retrieval, filtering, reprocessing, integration, and analysis [45]. However, the common problems in meta-analysis exist and are challenging. Identification of proper studies for meta-analysis is a time-consuming process as experimental information is often stored in a free-text format. The completeness and correctness of information largely depend on the thoroughness of the authors, and this issue constitutes a major challenge for microarray meta-analysis. Recently, there are a few published works on the meta-analyses of nasopharyngeal carcinoma using microarrays. Chen and collaborators combined the bioinformatics with evidence from biological experiments as a new way to gain more insights into the molecular mechanism of EBV-regulated neoplastic transformation [46]. By using a meta-analysis approach, they separated the sample into 2 metasets. The meta-A set was meta-analyzed to identify gene commonly activated or deactivated on EBV infection/reactivation in NPC (EBV reactivation in NPC versus EBV+/EBV—NPC). The meta-B set was meta-analyzed to obtain differentially expressed genes that are common in NPC and primary effusion lymphoma or PEL (EBV+/EBV—NPC versus EBV+/EBV—PEL). The meta-A and meta-B analyses revealed 23 and 45 differentially expressed genes, respectively. Then they integrated meta-A, meta-B, and related transcription factors into an interaction network using acquired information. A network of 23 meta-A genes in EBV-infected cells linked by some related transcription factors, of which the main nodes involve transcription factors JUN, CD9 and HOXA9. The 45 genes of meta-B network are connected by few related transcription factors CDKNIA, NFKBI, and MYC. The genes in meta-A and meta-B sets have been mapped into connected regulatory networks. There are 3 common genes between 2 sets including DEK, ITGA6, and DUSP1 [46]. Moreover, the regulatory network of genes involved in the EBV-dependent NPC reveals that NPC transformation depends timely on the regulation of DEK, CDK inhibitor, p53, RB, and several transcriptional cascades, which are interrelated by E2F, AP-1, NK-*κ*B, and STAT3 among others during latent and lytic cycles [46]. The meta-analysis of EBV-related tumor data may lead to further understanding of the EBV-related neoplastic transformation.

## 4. MicroRNA Omics

MicroRNAs (miRNAs) are a family of small noncoding nucleotide sequences which are able to complementarily bind to and negatively regulate gene expression at the posttranscriptional level, leading to either mRNA degradation or translational repression [47]. Primary miRNAs are usually transcribed from introns or noncoding regions and are cleaved in the nucleus by Drosha enzyme to yield hairpin precursor miRNAs (pre-miRNAs). Pre-miRNAs are then translocated into the cytoplasm and are subsequently cleaved by RNase III Dicer, giving rise to miRNA. These miRNA fragments execute their regulatory role as element of the RNA-induced silencing complex (RISC) [48, 49]. Research on miRNA as cancer biomarkers has gain considerable attention as miRNAs have been shown to play a role in fundamental cellular processes including cell proliferation and cell death and negatively control the expression of several cancer promoting proteins. In contrast to other types of molecular markers, miRNAs are relatively stable in the body and tissues, rendering them better candidates for cancer biomarkers [50].

A recent study investigated the miRNA expression profiles of two poorly differentiated NPC cell lines, CNE-2 and 6–10B, and their radioresistant sublines using next-generation deep sequencing [51]. Together with qRT-PCR validation, 3 downregulated miRNAs including miR-324-3p, miR-93-3p, and miR-4501, 3 upregulated miRNAs including miR-371a-5p, miR-34c-5p, and miR-1323, and 2 novel miRNAs have been identified to play a role in NPC radioresistance. One of the downstream targets for miR-324-3p is WNT2B, which has been reported to participate in the mediated NPC radioresistance [52]. However, identification of other downstream targets of these miRNAs needs further investigations. Furthermore, miRNA expression profiles in 312 paraffin-embedded specimens of NPC and 18 specimens of noncancer nasopharyngitis have been assessed. A total of 41 miRNA were differentially expressed between NPC and noncancer counterparts. The authors proposed a signature of five miRNAs with a prognostic value in addition to the TNM staging system [53]. In another study, differentially expressed plasma miRNAs in NPC patients including miR-483-5p, miR-103, and miR-29a were identified by next-generation sequencing as potential prognostic markers for NPC [54].

## 5. Proteomics

Proteomics approaches have been applied to discover cancer biomarkers. In the early days, the gel-based assay, in which two-dimensional gel electrophoresis (2DE) is coupled with mass spectrometry (MS), is utilized to screen the proteins with differential abundance between samples of different conditions of interest [55]. However, the disadvantages of this method include the time and labor inefficiency as well as low recovery rate of proteins. The gel-independent assay, in which liquid chromatography (LC) is used to separate peptides/proteins instead of 2DE and is combined with MS for protein identification, has later gained popularity as it offers superior protein identification and quantitation [56]. In the cell culture model, several proteomics-based molecular markers have been identified in various experimental settings. Jiang and coworkers [57, 58] reported the differential proteomes of a poorly differentiated squamous NPC cell line, CNE2, upon the treatment with 12-O-tetradecanoylphorbol 13-acetate (TPA), a known potent carcinogen for NPC. The results revealed upregulation of triosephosphate isomerase (TPI1) and 14-3-3 protein sigma (SFN) as well as downregulation of reticulocalbin 1 precursor (RCN1), nucleophosmin (NPM1), mitochondrial matrix protein p1 precursor (HSPD1), and stathmin (STMN1) in CNE2 cells following TPA treatment. Another study analyzed the proteomic profiles of an EBV-associated NPC cell line, C666-1, and a normal NP cell line, NP69, which showed that annexin II and beta-2-tubulin were suppressed in NPC cells [59]. Validation with immunocytochemistry also revealed that the downregulation of annexin II was positively correlated with lymph node metastasis, pointing to its potential application as a prognostic factor for NPC [59]. The proteins linked to the radioresistant trait of the NPC cells have been identified through proteomics in two independent studies using a radioresistant subclone cell line (CNE2-IR) derived from NPC cell line CNE2 [60, 61]. Feng et al. found the reduced expression of 14-3-3*σ* and the increased expression of Maspin, GRP78, and Mn-SOD in CNE2-IR cells compared to the control CNE2. The results were confirmed by Western blot and immunohistochemistry, suggesting that these proteins could serve as predicting biomarkers for patient response to radiotherapy and their dysregulation might be involved in the radioresistance of NPC [60]. On the other hand, Li et al. identified 16 differentially expressed proteins including upregulation of Nm23 H1 and downregulation of annexin A3 in the radioresistant NPC cells [61]. The different observations may arise from the fact that these two studies may have two different radioresistant sublines of CNE2 cells. Another study using the highly differentiated CNE1 cells and its radioresistant CNE1-IR subline demonstrated that the elevated level of heat shock protein 27 (HSP27) might play a role in radioresistance [62]. Moreover, differential proteomics of the CNE-2 and its highly metastatic subclone, S-18, and the knockdown experiment also suggested that HSP27 plays an important role in cancer metastasis and the corresponding downstream molecules could be NF-*κ*B, MMP9, and MMP11 [63]. Therefore, HSP27 could serve as prognostic and therapeutic target.

Comparative proteomics has been performed to identify differential expression proteins between the EBV− and EBV+ NPC cells [64]. Upon the EBV infection, a total of 12 proteins were identified as being significantly upregulated and associated with (i) signal transduction including voltage-dependent anion-selective channel protein 1 (VDAC1), S100-A2, hsc-70 interacting protein (Hip-70), ubiquitin, TPT1-like protein, and 4F2 cell surface antigen; (ii) cytoskeleton formation including keratin-75, tubulin beta-8 chain B, and dynein light chain 1; (iii) metabolic pathways including l-lactate dehydrogenase B chain (LDH-B) and triosephosphate isomerase (TIM); and (iv) DNA bindings including high mobility group protein B1 (HMG-1) [64]. These proteins provide a hint on the EBV-related mechanisms of NPC carcinogenesis and pose as potential biomarkers for the interaction of NPC-EBV. As cancer cells usually secrete biomolecules to enhance their proliferation, reduce apoptosis, and invade immune system [65], a few studies on differential secretomes for NPC have attempted to identify the secreted proteins that might be useful as cancer biomarkers and therapeutic targets. The secreted proteomes of two NPC cell lines including NPC-TW02 and NPC-TW04 cell lines were analyzed and a total of 23 proteins retrieved in both cell lines. Validation with Western blotting and immunohistochemistry confirmed their results, which indicated that fibronectin, Mac-2 BP, and PAI-1 might be potential molecular markers for NPC diagnosis [66]. Other secretome studies [67–70] also identified a cohort of proteins that might be useful as NPC biomarkers including chloride intracellular channel 1 (CLIC1) and C-C motif chemokine 5 (CCL5).

Several studies combined the laser capture microdissection of NPC tissues and proteomic analysis to identify protein markers for NPC. RKIP, a member of the phosphatidylethanolamine-binding protein family, has been identified to be a NPC metastasis suppressor and its suppression has been associated with the aggressiveness through the activation of MAPK pathway [71, 72]. The expression of stathmin, 14-3-3, and annexin I in NPC tissues has been shown to be correlated with differentiation and/or metastatic potential of the NPC cells; thus the dysregulation of these proteins might play a role in NPC development [73]. Among all identified proteins, cathepsin D [74, 75], cytokeratin 18 [76], L-plastin, S100A9 [77], a stroma-associated protein periostin [78], galectin-1 [79], keratin-8, SFN, and stathmin-1 [75] have been suggested to be biomarkers for NPC differentiation, progression, and prognosis.

As human blood holds a large reservoir of proteins and provides a less invasive mean of analytes for diagnosis, differential serum proteomics have been performed to identify even slight changes of certain proteins, which could potentially be biomarkers for NPC. Serum amyloid A protein (SAA) has been identified to be useful for invigilating the recurrent NPC cases [80]. Elevated levels of blood coagulation-related proteins including plasma kallikrein (KLKB1) and thrombin-antithrombin III complex (TAT) have been observed in NPC and could provide a diagnostic value for NPC cases [81]. A glycoprotein component of fibrinogen FGA in the serum has also been associated with NPC [82]. Apart from the individual protein markers, the MS signatures of the serum proteome in normal controls and NPC patients at different stages [83–85] and NPC with different levels of radiosensitivity [86] have been shown to be distinct.

It has been shown that the ERBB signaling pathway is dysregulated in NPC [25, 26]. This pathway is known to be tightly regulated by phosphorylation and dephosphorylation. Ruan and collaborators [87] have attempted to identify the downstream proteins, which are affected by stimulation of epithelial growth factor (EGF), by evaluating the phosphoproteome of CNE2 cells. A total of 33 proteins were identified in CNE2 upon the treatment with EGF. Among the identified proteins, glutathione S-transferase P1 has been validated using Western immunoblotting and knockdown experiments and has been linked to drug resistant trait in NPC cells [87]. Mitochondrial proteomes of the NPC cell lines 5–8F and 6–10B have been compared in order to find a clue for molecular mechanism of NPC metastasis and biomarkers related to metastasis [88]. A total of 16 mitochondrial proteins including PRDX3 and SOD2 were identified and serve as potential biomarkers for NPC. As these proteins are involved in the cellular response to reactive oxygen species, their abnormal function would play a role in oxidative stress, which could in turn mediate NPC metastasis [88].

## 6. Metabolomics

Metabolomics is considered to be a relatively new field of omics that simultaneously monitors many hundreds and thousands of small molecule metabolites from biofluids and tissue samples [89]. In any given conditions, a concerted function of metabolic processes occurs within a cell, which is readily changing in different physiological conditions. Hence, metabolomics represents a biochemical footprint of a physiological state of a cell. Metabolic profiles can be measured using nuclear magnetic resonance (NMR) spectroscopy and MS-based assays coupled with gas chromatography (GC-MS) or liquid chromatography (LC-MS) [90]. Differential metabolomes between case and control samples will lead to a cohort of molecules that has potential for early diagnosis, therapy, and understanding of the pathogenesis of many diseases. The metabolomics for NPC is still in its infancy. Recently, the metabolites of sera samples from 40 normal controls and 39 NPC patients were analyzed to find novel metabolic biomarkers [91]. Three novel candidate biomarkers including glucose, glutamate, and pyroglutamate were identified with the high specificity, suggesting that glycolysis and glutamate metabolism are involved in NPC carcinogenesis. Further validation of these molecules is warranted with larger cohorts of patients to prove their usefulness in terms of diagnosis. Yi et al. [92] performed a GC-MS-based metabolic profiling of 402 serum samples from NPC patients and normal controls. Metabolites including glucose, linoleic acid, stearic acid, arachidonic acid, proline, b-hydroxybutyrate, and glycerol 1-hexadecanoate were shown to have high distinguishing power of NPC from the healthy controls. Moreover, the metabolic signatures of the NPC patients who received radiotherapy appeared to resemble those of the normal controls, pointing to the possibility of applying metabolomics in assessing therapeutic effects.

## 7. Concluding Remarks

The omics technology enables the high-throughput profiling in the levels of genomics, epigenomics, transcriptomics, proteomics, and metabolomics, which lead to the large amount of data and together with bioinformatic tools can retrieve novel biomarkers. Current omics research in NPC has been reviewed, focusing on the biomarker discovery. A large number of potential biomarkers for NPC related to various pathophysiological states have been identified. However, extensive validation of these molecules in a larger cohort and in a multicenter platform is essential to verify their usefulness as biomarkers. In the future, it will be challenging to integrate the vast amount of multiomics data to gain better understanding of molecular basis of this complex malignancy.
